# Iron Oxide Scale Formation Mechanism and Anti-Corrosion Technology from Induction Remelting of Boiler Coating in Waste Incineration Power Plant

**DOI:** 10.3390/molecules30030689

**Published:** 2025-02-04

**Authors:** Zuopeng Qu, Xinli Tian

**Affiliations:** 1 College of New Energy, North China Electric Power University, Beijing 102206, China; 2 Jiangsu Kehuan Innovative Material Co., Ltd., Huai’an 223005, China

**Keywords:** coating protection, induction remelting, iron oxide scale, tube burst, inhibition technology

## Abstract

High-frequency induction welding technology represents the development direction of the high-temperature corrosion protection technology for the heating surfaces of the boiler “four tubes”. However, when the high-frequency induction coil heats and remelts the coating on the tube’s outer wall, the tube’s inner wall is also heated, causing an iron oxide scale to form on the tube’s inner wall. When the remelting temperature rises and the temperature of the tube’s inner wall exceeds 580 °C, three layers of oxide films, FeO, Fe_3_O_4_, and Fe_2_O_3_ are arranged in sequence from the substrate surface of the tube’s inner wall to the outside, with a thickness ratio of approximately 1:10:100. From the XRD spectra of tube iron oxide scale, it can be seen that the oxidation of the tube. The skin is mainly composed of Fe_3_O_4_, with a certain amount of Fe_2_O_3_ and trace amounts of FeO. The iron in the diffraction peak originates from the metal matrix. However, when the remelting temperature continues to rise and the temperature of the tube’s inner wall exceeds 580 °C, the oxide film begins to thicken significantly, that is, the oxide film begins to transform into an oxide scale. Under the continuous action of high-temperature induction remelting, the reaction between iron and oxygen is accelerated, but because the oxygen ions of water slowly diffuse through two outer layers of oxide films, with a low oxygen concentration. Although the FeO film is thin, it has a loose structure and numerous lattice defects, is unstable and easy to decompose, and easily peels off from the tube’s inner wall. For a pipe wall thickness of 5 mm, if the thinning rate of the inner wall caused by detachment reaches 0.8 mm/year, it is highly likely to cause pipe burst accidents within 4–5 years. The influence of the iron oxide scale on the performance of the tube’s inner wall was evaluated by testing indexes, such as surface hardness and decarburization layer depth. Although the oxide scale reduces the surface hardness of the tube’s inner wall, the surface decarburization layer is very thin, so the effect on the mechanical properties of the tube’s substrate is limited. The technology of inhibiting the formation of the iron oxide scale in induction remelting is briefly introduced. During the high-frequency remelting process of water-cooled walls, as the tube bank moves forward relative to the high-frequency heating coil, nitrogen protection is used to suppress the formation of oxide scale, effectively eliminating the troubles caused by high-frequency induction remelting and achieving the goal of improving the service life of the tube bank. This technology of the nitrogen protection method is used to inhibit the formation of iron oxide scale, not only inhibiting the formation of the iron oxide scale on the tube inner wall and the back of the tube bundle, with remarkable experimental results and broad application prospects.

## 1. Introduction

China’s waste incineration power generation technology has developed for nearly 30 years. Especially after 2010, waste incineration power generation has begun to develop rapidly. In 2018, the waste incineration power plants ranked first in the world in terms of installed capacity and generating capacity. It was estimated that by the end of 2021, China’s installed power-generating capacity would reach 13.47 million kilowatts [1].

**Figure 1:**
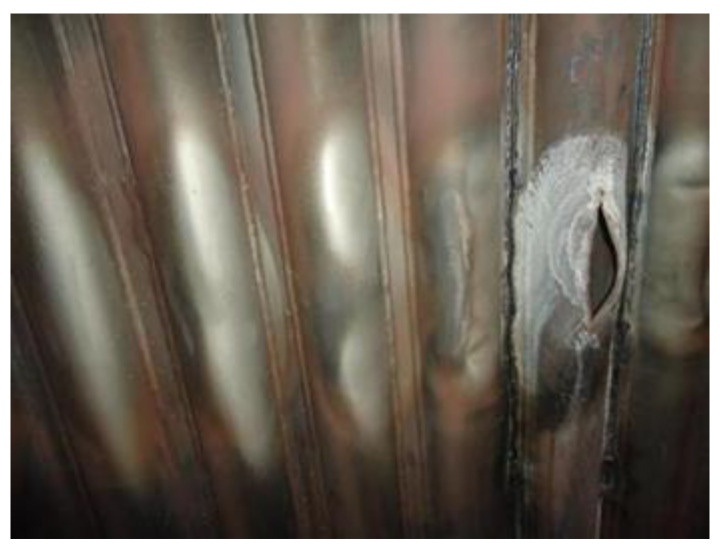
Macroscopic morphology of water wall tube burst.

**Figure 2:**
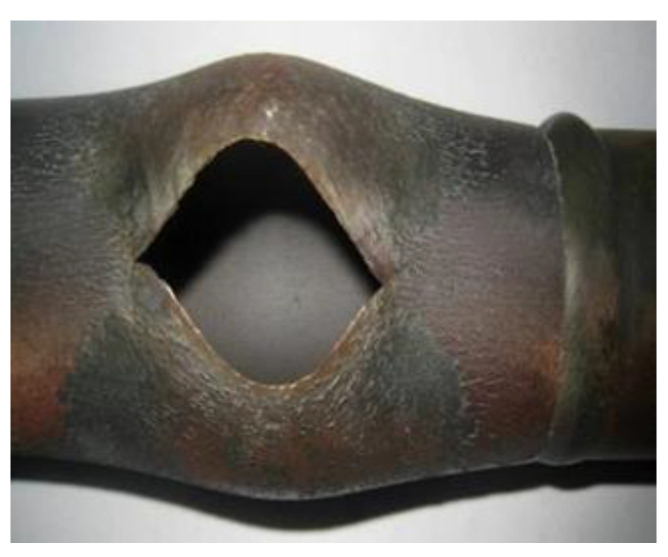
Macroscopic morphology of superheater tube burst.

Over the years, how to effectively control the high-temperature corrosion of the heating surface of the waste heat boiler while optimizing the steam parameters for power generation and improving the efficiency of waste incineration power generation has always been a hot topic of research for scholars at home and abroad. The water wall tube bundles and superheaters of the waste incineration power boilers are severely corroded at high temperatures, and chloride corrosion is dominant. This is because the composition of waste is complex and changeable, and chlorine, sulfur, alkali metals, and heavy metals experience complex interactions at high temperatures in the incinerator, easily forming compounds with low melting points and high vapor pressure, causing severe corrosion of the heating surfaces of the boiler “four tubes” (water wall, superheater, reheater, and economizer), and resulting in rapid thinning of the tube wall and frequent tube bursts (see Figure 1 and Figure 2). As a result, water incineration power plants frequently have to shut down for unscheduled maintenance, which not only affects waste treatment efficiency, but also brings high maintenance costs and huge economic losses. This problem has become a bottleneck hindering the technological development of the water incineration power generation industry. In recent years, regarding the high-temperature corrosion protection of boilers, many scholars have suggested improvements in furnace structure design, process control, tube wall material selection, etc., and have made certain progress [2]. However, since corrosion tends to occur on the material surface, an appropriate surface protection technology has been increasingly recognized in recent years as the most effective and reasonable means of protection and also has better economic benefits and maintenance convenience.

To improve the service life of pipelines, coating protection is relatively cost-effective. A variety of coating technologies have been explored for high-temperature corrosion protection both at home and abroad for many years. Traditional thermal spraying techniques such as flame spraying, arc spraying, plasma spraying, etc., have a low bonding strength and a protection life of no more than 2–3 years, while supersonic flame spraying (HVOF) can be extended by 1–2 years. Laser cladding coatings have been fired for a period of time, but due to the tendency of cracking in the cladding layer and the difficulty in adjusting laser parameters, they have not been widely applied [3]. As a rule, the method of Inconel 625 alloys surfacing on the heating surface of boiler tubes is generally adopted to improve their corrosion resistance and prolong the service life of these tubes. Although this method has been used for years and its overall effect has been recognized by the industry, some problems are emerging. Among them, the biggest problem is that sensitization will occur in the material at 450 °C, that is, only if beyond this temperature, the corrosion resistance of the material will drop rapidly, and it is difficult to meet the requirement for the service life of 6–8 years. Especially with the increasingly rapid development of high-parameter boilers, the surfacing method has been increasingly unable to meet the needs. To address the problem of the high dilution rate of the substrate, the thickness of the surfacing layer must exceed 2.0 mm, which brings about problems such as low efficiency and high cost. Laser cladding is a surface technology developed in recent years. Compared with surfacing technology, laser cladding technology has less thermal impact on the base metal and can effectively solve the problem of tube welding deformation. However, some problems still limit its practical application, such as high equipment input and complex construction technology. Hence, this technology is still research-based in this field and has not been put into practical application till now [4].

**Figure 3:**
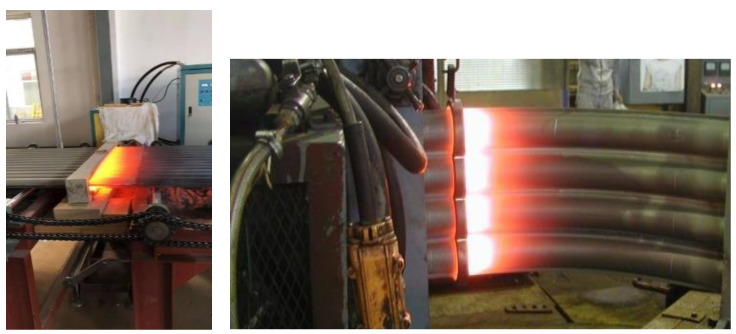
High-frequency induction remelting of water wall tube bundle.

High-frequency induction welding technology is a relatively new technology for surface protection. Japan successfully used this technology for high-temperature corrosion protection of waste incineration power boilers in 2009, with remarkable results. Taiwan introduced this technology from Japan in 2011 and achieved good results. This technology has now been popularized and applied in waste incineration power plants throughout Taiwan Island [4]. This research team has taken the lead in developing this technology in China since 2016, and the composite method of flame-sprayed nickel-based self-fluxing alloy and high-frequency induction remelting has been successfully applied in the technology of preparing the coating on the heating surface of the boiler water wall tube bundle. The induction welding process of the water wall tube bundle is shown in Figure 3. This method is more advanced than the traditional surfacing method, which is mainly reflected in that the corrosion resistance of the induction remelting coating of the nickel-based self-fluxing alloy will not decrease when the temperature exceeds 450 °C, and a thick coating (>2 mm) for surfacing is replaced with a thin coating (approximately 0.5 mm), which significantly improves the coating preparation efficiency and greatly reduces the cost. After several years of application practice, this technology has gained a good reputation in the waste incineration power plant industry; that is, it represents the development direction of the technology for high-temperature anti-corrosion on the heating surface of the boiler water wall tube bundle.

## 2. Formation of Oxide Scale and Its Formation Mechanism

### 2.1. Formation of Iron Oxide Scale During Remelting

**Figure 4:**
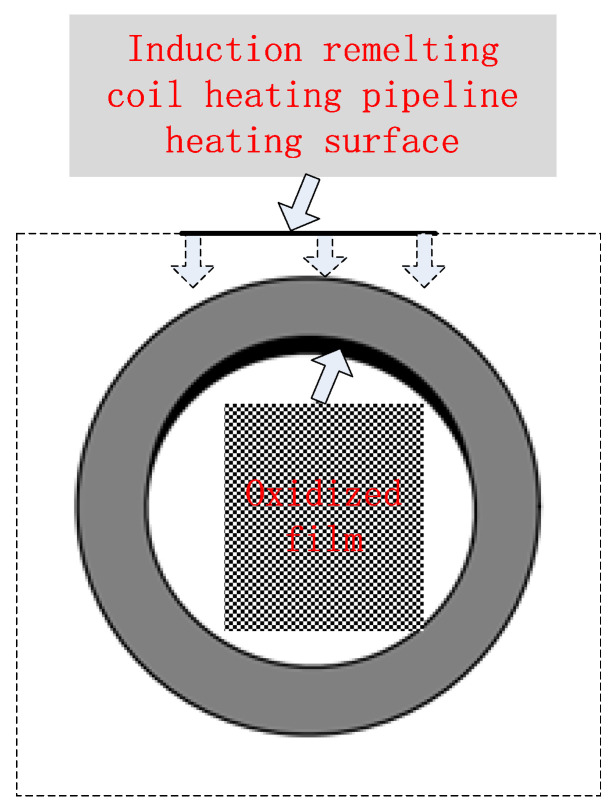
Schematic diagram of oxide film formed on the tube’s inner wall during induction remelting.

**Figure 5:**
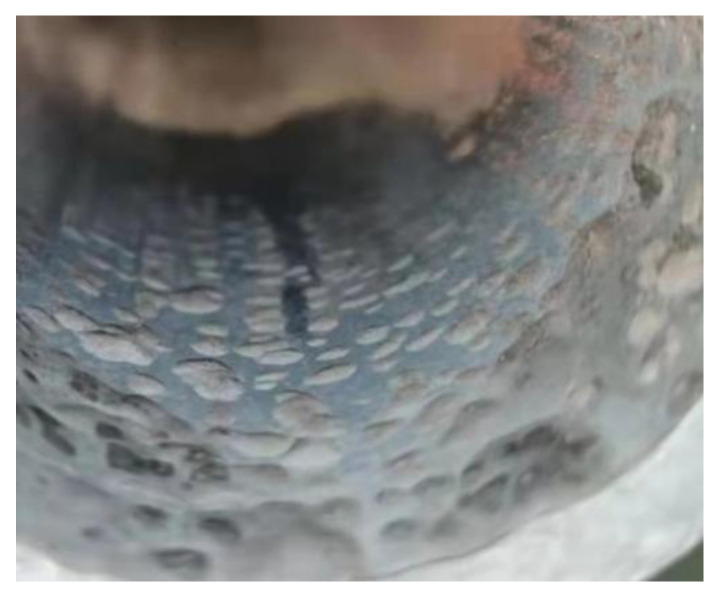
Iron oxide scale formed on the tube’s inner wall during remelting.

**Figure 6:**
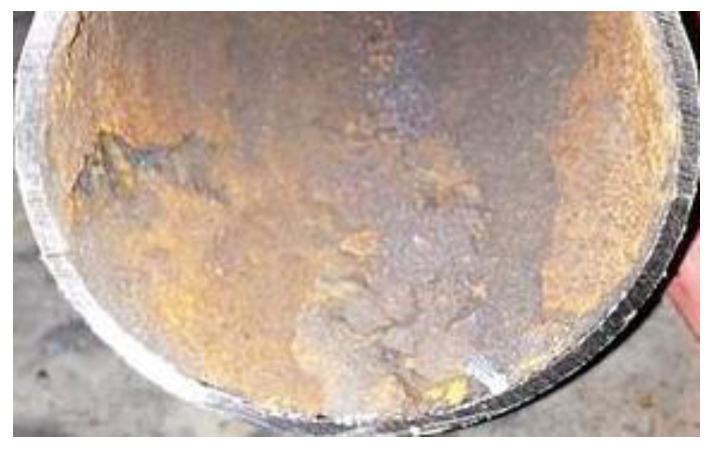
Iron oxide scale on the tube’s inner wall after remelting and one year of service.

Although the high-frequency induction welding method has a significant high-temperature anti-corrosion effect, there exists trouble, that is, the high-frequency induction coil heats and remelts the heating surface coating at approximately 1050 °C. While the coating is heated, rapid heat conduction of the metal steel tube allows the tube’s inner wall to be heated. High-frequency induction coil heating has a skin effect, that is, the heating temperature of the coating closest to the inner surface of the coil is the maximum, but the temperature gradient is large, and the temperature drops rapidly during the conduction from the outer wall to the inner wall along the tube wall thickness. When the heat is conducted to the tube’s inner surface, the temperature detected by an infrared detector is lower than 800 °C, but under which high-temperature oxidation and formation of oxide film on the tube’s inner wall surface are prone to occur [5]. Figure 4 is a schematic diagram for the formation of the oxide film on the tube’s inner wall during induction remelting. Figure 5 shows local oxidation for the tube’s inner wall remelting. It can be seen from Figure 5 that the iron element in the metal on the tube’s inner wall after induction remelting reacts with oxygen in the air at high temperatures, and the resulting iron oxide scale is locally scaly. Figure 6 shows the macroscopic morphology of the iron oxide scale on the tube’s inner wall after remelting and one year of service. It can be seen that after a certain time of service, the iron oxide scale on the tube’s inner wall has been connected into pieces under continuous oxidation of water vapor, with a certain thickness, but uneven; the thickest part is 0.8 mm.

### 2.2. Formation Mechanism of Oxide Scale

**Figure 7:**
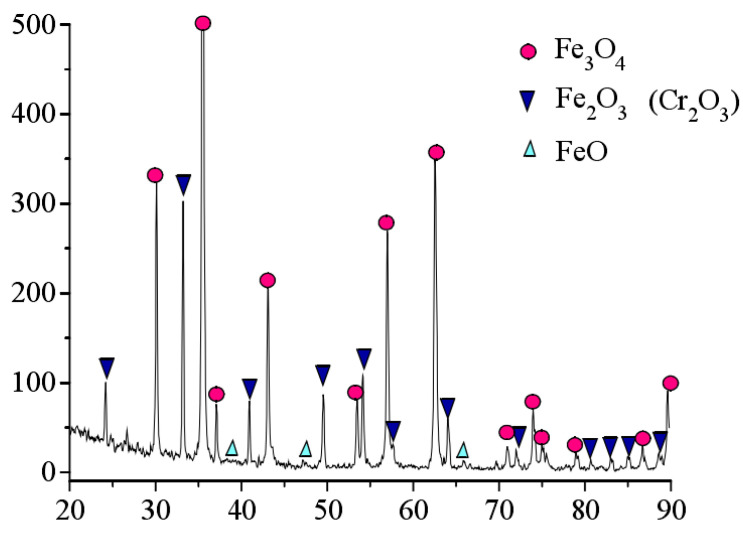
XRD spectra of the TP347H tube iron oxide scale.

Theoretically, the reason for the formation of oxide film on the tube’s inner wall is that under high-temperature heating of the induction coil on the tube’s outer wall, the oxidation of iron ions, a main element of the tube’s material, is completed through the continuous diffusion of oxygen ions to the tube’s inner wall. When induction remelting begins, the oxide film is composed of Fe_2_O_3_ and Fe_3_O_4_ generally at below 580 °C, but the crystal structures of Fe_2_O_3_ and Fe_3_O_4_ are relatively dense, thus protecting the tube’s inner wall from further oxidation. Figure 7 shows the XRD spectrum of the iron oxide scale of the TP347H tube (the horizontal axis represents diffraction angle, and the vertical axis represents spectral line intensity).

**Figure 8:**
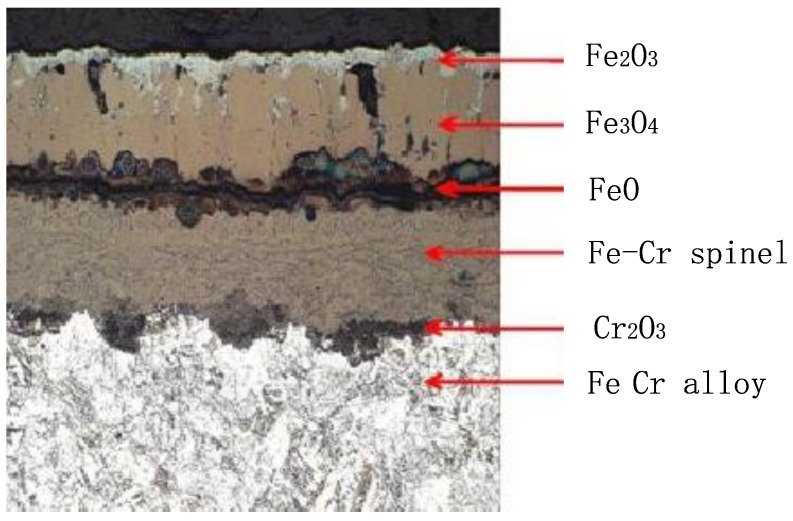
High temperature promotes the large-scale production of interstitial iron ions.

From Figure 7, it can be seen that the oxidation of the TP347H tube. The skin is mainly composed of Fe_3_O_4_, with a certain amount of Fe_2_O_3_ and trace amounts of FeO. The iron in the diffraction peak originates from the metal matrix. However, when the remelting temperature continues to rise and the temperature of the tube’s inner wall exceeds 580 °C, the oxide film begins to thicken significantly, that is, the oxide film begins to transform into an oxide scale. Under the continuous action of high-temperature induction remelting, the reaction between iron and oxygen is accelerated, but because the oxygen ions of water slowly diffuse through two outer layers of oxide films, with a low oxygen concentration. At this time, iron and oxygen will react with FeO under over-temperature conditions. Figure 8 shows that when the temperature inside the T91 metal pipeline exceeds 610 °C, unstable FeO will appear in the inner layer, which is also important evidence that high temperature promotes the large-scale production of interstitial iron ions under low oxygen partial pressure conditions. The double-layer oxide film on the tube’s inner wall will become a triple-layer oxide film, which is FeO, Fe_3_O_4_, and Fe_2_O_3_ from the substrate surface of the tube’s inner wall to the outside, with a thickness ratio of approximately 1:10:100 [6].

As the service time of the boiler increases, the iron oxide scale on the tube’s inner wall gradually thickens. Assuming that the boiler running time is 2.05 × 10^5^ h, some scholars have statistically shown that the accumulated oxide thickness at the steam temperature of 650 °C in the tube is shown in Table 1 [7]. The FeO layer is directly connected to the tube’s inner wall substrate. Although the FeO layer is the thinnest, the instability of the iron oxide scale is caused by loose structure, many lattice defects, and easy decomposition. Moreover, the formation of the oxide scale in the innermost layer easily causes the entire oxide scale to peel off from the tube’s inner wall. Due to the difference in thermal expansion coefficients between the oxide layers of the iron oxide scale and the tube substrate (taking the T23 steel tube as an example, as shown in Table 2 [7]), the iron oxide scale is easily cracked and peeled off under the continuous action of thermal stress generated by high-pressure steam in the tube during the long-term service of the boiler.

**Figure 9:**
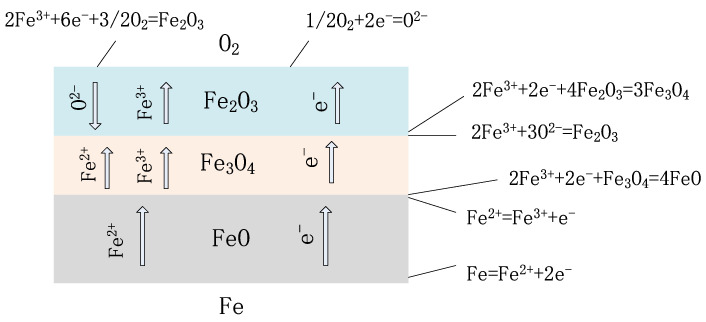
Schematic diagram of the formation mechanism of oxide film under high-temperature remelting [8].

The structure of the whole iron oxide scale on the tube’s inner wall caused by high-temperature remelting is shown in Figure 9 [8]. As shown in Figure 9, in the iron-oxide layer-oxygen system, there are two interfaces: the iron-oxide layer interface and the oxide layer-oxygen interface. To keep the reaction continuing, the iron element should migrate through the oxide layer to the oxide layer-gas interface where it reacts, or oxygen should migrate to the oxide layer-iron interface where it reacts. Thus, the penetration of the reactant through the iron oxide layer is the core of the mechanism of high-temperature oxidation on the tube’s inner wall.

Domestic and foreign studies have found that the iron oxide scales of most waste incineration boilers peel off after a period of time, and the formation time of the iron oxide scale is different on different steels. Table 3 provides the statistics of boiler iron oxide scale peel-off in some thermal power units in the United States [9].

According to professors IGWright and BAPint of the United States, the thickness D of the iron oxide scale formed during high-temperature oxidation follows the following rule [9]:

(1) $$D=K_{p}\cdot t^{n}=A\cdot e^{-Q/(RT)}\cdot t^{n}$$

where *K_p_*—oxidation rate constant; *Q*—activation energy of metal oxidation (J/mol); *T*—metal temperature (*K*); t—running time (h); *A*—Arrhenius constant; *R*—gas constant; *n*—index number.

According to Formula (1), the influence of metal oxidation activation energy *Q* on the thickness of the iron oxide scale is crucial. Taking low-alloy steel tubes containing alloy materials such as Cr and Mo, widely used in water wall and superheater tubes, as an example, if the base metal of the tube contains 0–2% Cr alloy, the activation energy *Q* of the oxide scale is divided at 500 °C: if below 500 °C, the activation energy of the iron oxide scale is 85 kJ/mol; if above 500 °C, the activation energy of the iron oxide scale is 235 kJ/mol. Therefore, when the temperature is higher than 500 °C, the formation rate of the iron oxide scale will greatly increase. For alloy tubes containing 9–12% Cr, such as the common boiler superheater tube 12 Cr1MoVG, the activation energy of the iron oxide scale is approximately 146 kJ/mol within 550–700 °C. Therefore, the temperature has a great influence on the thickness of the iron oxide scale; at the same temperature, the iron oxide scales formed on the inner walls of the tubes composed of different materials have different thicknesses.

**Figure 10:**
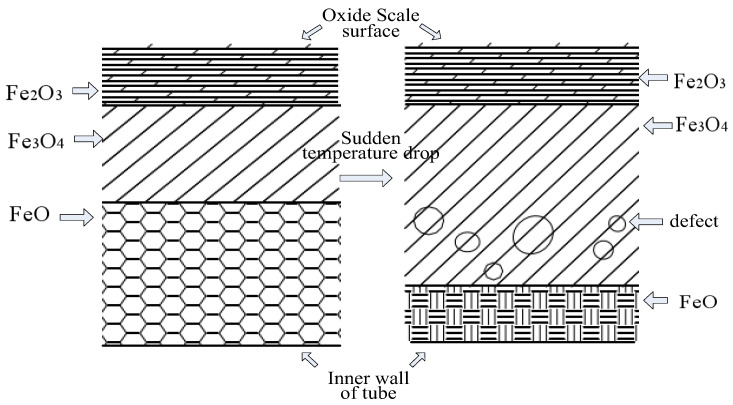
Schematic diagram of the impact of element diffusion and sudden temperature drop on oxide scale.

During the induction remelting process on the outer wall of the tube, the 2-layer oxide film on the metal surface inside the tube will turn into a 3-layer oxide film, starting from the inner wall surface of the tube and extending outward in sequence as FeO, Fe_3_O_4_, Fe_2_O_3_. The inner FeO layer is connected to the metal substrate, as shown in Figure 10. Due to the loose structure of the inner layer FeO, there are many lattice defects and instability. When the temperature drops sharply after induction remelting, the inner FeO layer will decompose into Fe_3_O_4_ and Fe, making the Fe3O4 layer loose, as shown in Figure 10. From Figure 10, it can be seen that after the temperature decreases, the inner oxide film will decompose, and defects will appear during the decomposition process of the inner oxide skin, resulting in a porous and loose interface between the intermediate oxide skin and the (Fe, Cr)_3_O_4_ spinel structure inner oxide, which creates a prerequisite for the peeling of the oxide scale.

### 2.3. Hazards of Oxide Scale Peel-Off

Due to the huge difference in thermal expansion coefficients between the tube’s substrate and the iron oxide scale (see Table 2) on the tube’s inner wall, when the boiler is frequently started and stopped during service, the metal tube and the iron oxide scale on the surface are both subject to thermal expansion and contraction, and the iron oxide scale will gradually peel off from the tube’s inner wall.

The decrease in the tube diameter after the iron oxide scale peels off will affect the service life of the tube bundle, with a more serious impact inside the tube. Oxide scale peel-off from high-temperature parts such as boiler superheaters, reheaters, and main steam tubes will cause the following problems and serious consequences [10]:

It leads to the blockage of boiler tube bends, which causes local overheating of the metal heating surface of the boiler and even tube bursts. During the startup and shutdown of the unit, the oxide peel-off rate is the maximum. Part of the oxide scale peeled off is carried out of the water wall, superheater, and reheater with the high-speed flowing steam, and finally trapped at the turning point. When the iron oxide scale has accumulated to a certain thickness, significant overheating of the tube wall at the turning point will cause the tube to burst.

① The entry of the oxide scale peeled off into the turbine will damage the flow passage of the steam turbine, and these oxide scale particles have great kinetic energy, in which case the nozzle and blades of the turbine are continuously hit, which not only causes great damage to the nozzle and blades of the turbine, but also shortens the maintenance cycle and increases the maintenance cost.

② If the debris of the oxide scale peeled off is deposited between the valve core and the valve seat of the turbine main valve, the turbine main valve may be stuck. If the main steam valve is not tightly closed during load shedding, this will cause turbine overspeed and affect the safe running of the unit.

## 3. Influence of Oxide Scale on Mechanical Properties of Tube Inner Wall

Under high-temperature remelting of the high-frequency induction coil, the iron element on the tube’s inner wall will oxidize with the air. The influence of the reactant on the mechanical properties of the tube’s inner wall is mainly detected based on two indicators of surface hardness and decarburization layer depth.

### 3.1. Surface Hardness

**Figure 11:**
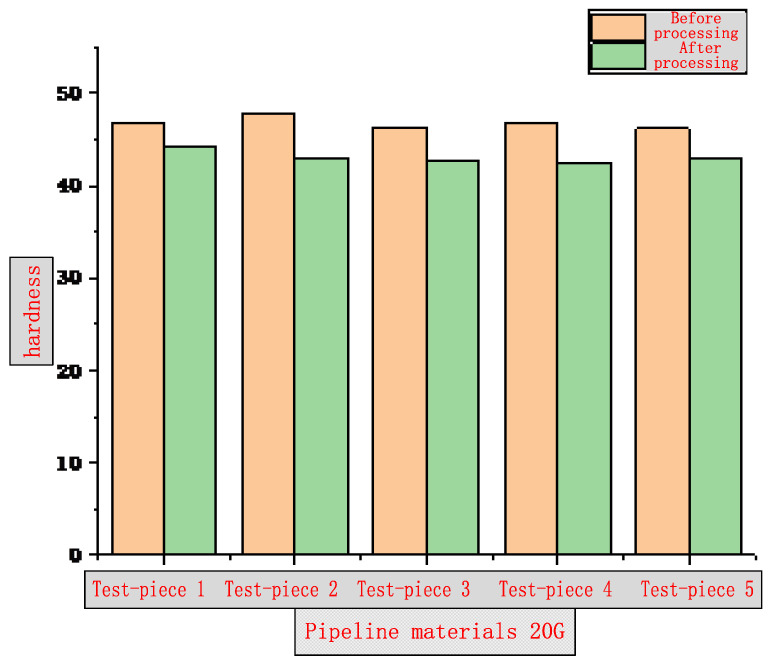
Diagram of 20 G hardness test results.

**Figure 12:**
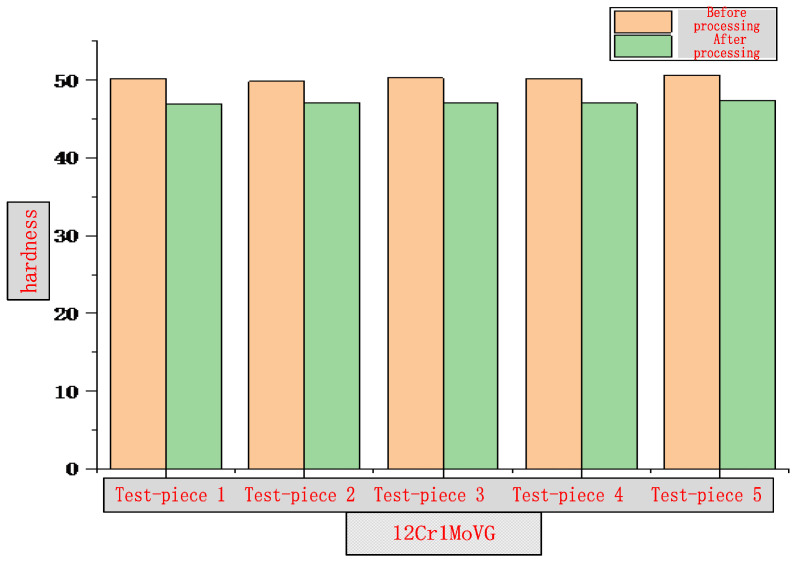
Diagram of 12 Cr1MoVG hardness test results.

The surface hardness of the inner wall of two common water wall and superheater tube specimens, namely 20 G and 12 Cr1MoVG, after induction remelting and heating, was tested by Rockwell hardness test. According to the requirements of GB/T 230.1-2018, the specimens were fixed on the workbench of the hardness tester at a temperature of 23 ± 5 °C and a humidity of 40–80%RH [11]. A diamond cone indenter was pressed into the specimen surface at a pressure of 98.7 N to measure the initial indentation depth, and then the main pressure of 588.4 N was applied. After the main pressure was relieved, the indentation depth was measured again, and the Rockwell hardness was calculated based on the difference in indentation depths. A total of five specimens were measured. The test results are shown in Figure 11 and Figure 12. Compared with the samples not subjected to induction remelting, the surface hardness of the samples after induction remelting is reduced. However, the change rate is 5.35–9.86% for 20 G tube specimens and 5.43–6.58% for 12 Cr1MoVG tube specimens, indicating that the surface hardness of the two has decreased, mainly because the iron oxide scale is formed on the tube inner wall at approximately 800 °C after remelting, leading to a decrease in the density compared with the substrate material. As a result, the surface hardness decreases by less than 10%. The influence of the decrease in surface hardness on the application performance should be comprehensively assessed based on the investigation results of the decarburization layer thickness.

### 3.2. Decarburization Layer Depth Test

To further investigate the reasons for the decrease in the hardness of the tube’s inner wall and determine whether the performance of the tube substrate is affected, a relatively simple method to detect the decarburization layer depth on the surface of the tube substrate is performed. Considering that the 12 Cr1MoVG tube is obviously superior to the 20 G tube, it is acceptable that only the latter is tested. When the inner wall of the 20 G water tube is heated by an induction remelting coil, the tube inner wall is very likely to partially or completely decarbonize under the oxidation effect, so that the mass fraction of the surface carbon is lower than that of the interior. Theoretically, the hardness decreases as the porosity in the decarburization layer increases.

**Figure 13:**
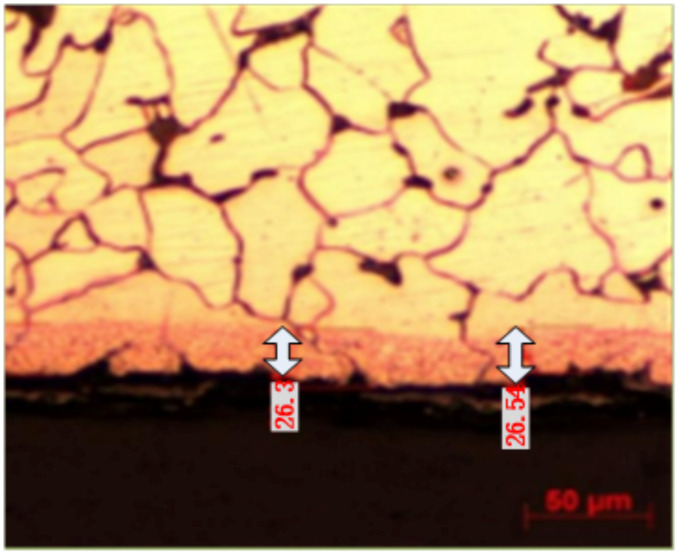
Depth of complete decarburization layer of the tube’s inner wall.

According to the china national standard, samples were cut from the inner surface of the 20 G tube sample at a temperature of 23 ± 5 °C and a humidity of 40–80%RH [12]. The depth of the complete decarburization layer was tested using the metallographic method. Five random measurements were taken within a microscope field of view in a uniform decarburization zone, and the average value was taken. The test results are shown in Table 4, and Figure 13 shows the depth of the complete decarburization layer on the tube’s inner wall.

As can be seen from Table 4 and Figure 13, according to the requirements of GB/T 5310-2017 for the depth of the complete decarburization layer of a 20 G steel tube [14], the depth of the surface decarburization layer should not be greater than 0.4 mm, while the actual sample decarburization layer depth is less than 0.03 mm, fully in line with the use requirements. A very small depth of the decarburization layer on the surface of the tube after induction remelting is directly related to high-frequency induction heating. Since this heat source has a skin effect [15], the maximum temperature is only concentrated in a very thin layer on the outer wall, and a large temperature gradient leads to a rapid temperature drop toward the tube’s inner wall. Hence, the temperature of the tube’s inner wall drops rapidly before decarburization.

The test results show that, after the outer wall of the 20 G tube has been remelted, an oxide scale is formed on the inner wall. Although the surface hardness is reduced, the thickness of the surface decarburization layer is very small, so the mechanical properties of the tube substrate are affected to a limited extent.

## 4. Technology of Inhibiting Tube Bundle Oxidization Depending on Coating Induction Remelting

To deal with the serious consequences of tube bursts resulting from iron oxide scale peel-off, this research team has proposed a technology of inhibiting tube oxidation depending on gas protection during high-frequency remelting of the surface coating of the boiler “four tubes”. This technology was granted a Chinese invention patent (ZL201910731494.3) in January 2021. Taking the water wall as an example, as the tube bundle moves forward relative to the high-frequency heating coil during high-frequency remelting, the nitrogen protection method is used to suppress the formation of iron oxide scale, effectively eliminating the troubles caused by high-frequency induction remelting and prolonging the service life of the tube bundle.

Specifically, as the tube bundle moves forward relative to the high-frequency heating coil during high-frequency remelting of the boiler water wall, the nitrogen exhaust method is used to isolate from the oxygen in the air inside the tube and on the back of the tube bundle, thereby inhibiting the formation of iron oxide scale. Among them, the method for inhibiting the iron oxide scale inside the tube bundle is as follows: seal the tube openings at both ends of the tube bundle with a sealing plate, drill an internal threaded hole in the sealing plate, screw a ceramic nozzle with an external thread into the internal threaded hole of the sealing plate, and continuously spray high-pressure nitrogen into the tube. Nitrogen is a common protective gas because it is inert and difficult to react chemically. The process of introducing nitrogen into the tube is actually a process of exhausting air. Without oxygen or at a very low concentration of oxygen, the formation of the oxide film on the tube’s inner wall can be avoided during high-temperature remelting.

**Figure 14:**
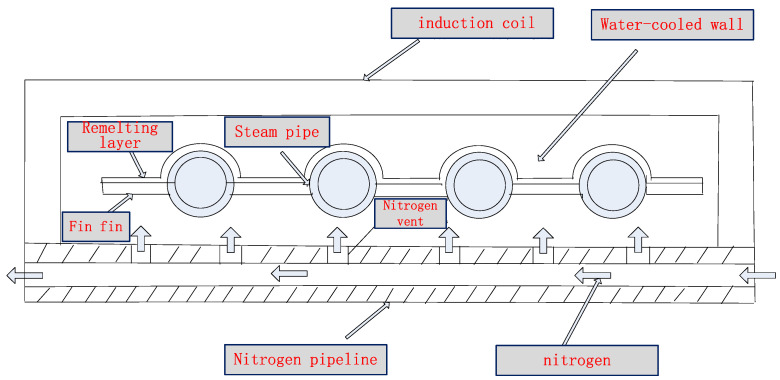
Schematic diagram of the technical solution for inhibiting oxidation on the tube’s inner wall and back of the tube bundle.

The iron oxide scale on the back of the tube bundle cannot be inhibited without nitrogen protection, because fatigue cracks are prone to occur on the back of the oxidized fin, which will cause perforation over time. Corrosive gases enter the back of the tube bundle and then corrode the inner wall of the boiler. Furthermore, the supporting strength of the tube bundle decreases, causing the tube bundle to partially tilt or deform and posing a safety hazard. The technical solution is shown in Figure 14. A copper tube for water cooling of the outer layer is erected and fixed in parallel with the high-frequency induction copper tube coil and close to the bottom of the coil. Nitrogen is introduced through a copper tube, and a row of small holes is drilled above the copper tube with a certain interval. Nitrogen is sprayed out with a specific pressure and flow rate to inhibit oxidation on the back of the tube bundle.

**Figure 15:**
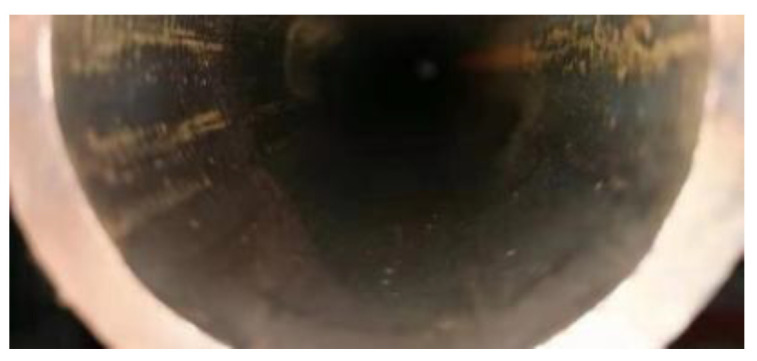
Unremelted tube’s inner wall.

**Figure 16:**
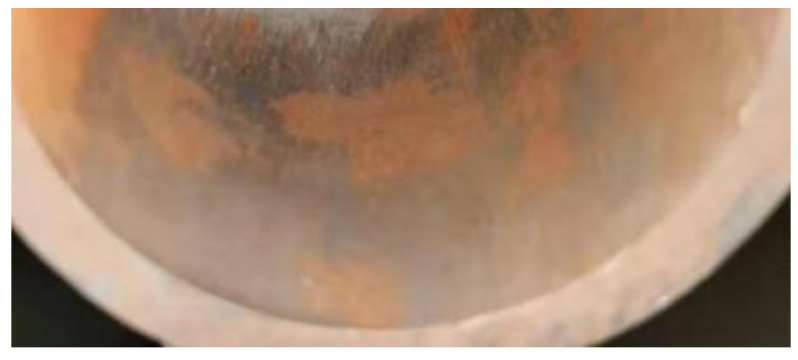
Tube’s inner wall after nitrogen protection.

Experiments have shown that by continuously blowing a small amount of nitrogen into the tube bundle and the back, the formation of iron oxide scale inside the tubes and on the back of the tube bundle can be effectively inhibited even during remelting and high-temperature baking. This method can not only effectively eliminate the potential risk of tube burst caused by oxide scale peel-off, but also improve the fatigue life of the tube bundle substrate material. In terms of economic performance, the market price of industrial nitrogen is approximately RMB 35/bottle. Benefiting from its small flow rate and low consumption, remelting of the tube bundle per 9 m consumes no more than 4 bottles, costing approximately RMB 140. By testing and optimizing different inert gases, pressures, flow rates, and process parameters of the nozzle and dynamic sealing structure, it is determined that the parameters such as nitrogen pressure of 0.5–0.7 Mpa and flow rate of 0.7–1.7 m^3^/h are used according to different inner diameters of the tube bundle. It can be seen from Figure 5, Figure 15 and Figure 16 that this method has a significant effect on inhibiting the formation of iron oxide scale inside the tube during high-frequency induction welding, indicating that this method has a high cost-effectiveness and also proves that this technology has good application prospects. The application of this technology has now begun to be promoted.

## 5. Conclusions

(1) When the remelting temperature is below 570 °C, the oxide film on the tube’s inner wall is only composed of Fe_2_O_3_ and Fe_3_O_4_, and the crystal structures of Fe_2_O_3_ and Fe_3_O_4_ are relatively dense, thus protecting the tube’s inner wall from further oxidation. When the remelting temperature rises and the temperature of the tube’s inner wall exceeds 570 °C, an oxide scale is formed of three layers of oxide films of different thicknesses, namely FeO, Fe_3_O_4_, and Fe_2_O_3_, extending from the substrate surface of the tube’s inner wall outward, with a thickness ratio of approximately 1:10:100.

(2) The effect of the induction remelting process on the mechanical properties of the tube’s inner wall is assessed by testing the surface hardness and the decarburization layer depth. Although the oxide scale reduces the surface hardness of the tube’s inner wall, the surface decarburization layer is very thin, so the effect on the mechanical properties of the tube substrate is limited.

(3) The core of the technology of inhibiting tube oxidation depending on gas protection during remelting is that during the high-frequency remelting of the water wall, as the tube bundle moves forward relative to the high-frequency heating coil, the nitrogen protection method is used to inhibit the formation of iron oxide scale, effectively eliminating the troubles caused by high-frequency induction remelting and prolonging the service life of the tube bundle, with remarkable experimental results and broad application prospects.

## Figures and Tables

**Table 1:** Cumulative Thickness of Iron Oxide Scale on the Inner Wall of the Tubes Composed of Various Materials after 2.05 × 10^5^ h [7].

Alloy Material	Temperature Range (°C)	Oxide Scale Thickness (μm)
0–2%Cr	500–700	520
9–12%Cr	450–700	140
0–2%Cr	470–1200	2307
0–2%Cr	450–1200	603

**Table 2:** Linear Expansion Coefficient of T23 Metal Substrate and Oxide (×10^−6^ K^−1^) [7].

Temperature (°C)	100	200	300	400	500	600	700
T23	17.10	17.40	17.80	18.30	18.90	19.10	19.40
Fe_3_O_4_	-	-	-	-	9.10	-	-
Fe_2_O_3_	-	-	-	-	14.90	-	-
FeO	-	-	-	-	12.20	-	-

**Table 3:** Statistics on Iron Oxide Scale on Different Steels in Use in the United States [9].

Piping Material	Cr (%)	Power Generation Temperature (°C)	Detachment Time (h)	Thickness of Oxide Film (μm)
T91	8–9.5	600–605	11,000	330
E911	8.5–9.5	600–605	11,000	460
NF616	8.5–9.5	545	10,149	105
NF616	8.5–9.5	600–605	11,000	325
HCM12	11–13	545	10,149	60
Esshetc 1250	14–16	600	12,463	40–80
Esshetc 1250	14–16	660	12,719	100–148
Esshetc 1250	14–16	633–677	6840	200–230
Super 304H	17–19	565	10,149	40–50
NF709	19–22	565	10,149	20–25
HR3C	24–26	565	10,149	30
AC66	26–28	600	12,463	-

**Table 4:** Test Results of Complete Decarburization Layer Depth of Inner Wall after 20 G Sample Treatment.

Position 1 (μm)	Position 2 (μm)	Position 3 (μm)	Position 4 (μm)	Position 5 (μm)	Average (μm)
GB/T 5310-2017 [13] 20 G Depth Requirement of Complete Decarburization Layer: Inner Surface < 400 μm					
22.07	23.39	23.17	26.01	20.54	23.04

## Data Availability

Data is contained within the article.
